# Nanomaterials for Delivering Antibiotics in the Therapy of Pneumonia

**DOI:** 10.3390/ijms232415738

**Published:** 2022-12-12

**Authors:** Jie Tang, Qiuhong Ouyang, Yanyan Li, Peisen Zhang, Weihua Jin, Shuang Qu, Fengmei Yang, Zhanlong He, Meng Qin

**Affiliations:** 1 Institute of Medical Biology, Chinese Academy of Medical Sciences & Peking Union Medical College, Kunming 650118, China; 2 College of Life Science and Technology, Beijing University of Chemical Technology, Beijing 100029, China

**Keywords:** bacterial pneumonia, traditional antibiotic therapy, bioactive nanoparticles, pulmonary inhalation

## Abstract

Bacterial pneumonia is one of the leading causes of death worldwide and exerts a significant burden on health-care resources. Antibiotics have long been used as first-line drugs for the treatment of bacterial pneumonia. However, antibiotic therapy and traditional antibiotic delivery are associated with important challenges, including drug resistance, low bioavailability, and adverse side effects; the existence of physiological barriers further hampers treatment. Fortunately, these limitations may be overcome by the application of nanotechnology, which can facilitate drug delivery while improving drug stability and bioavailability. This review summarizes the challenges facing the treatment of bacterial pneumonia and also highlights the types of nanoparticles that can be used for antibiotic delivery. This review places a special focus on the state-of-the-art in nanomaterial-based approaches to the delivery of antibiotics for the treatment of pneumonia.

## 1. Introduction

Pneumonia is one of the leading causes of death worldwide [1]. Pneumonia accounted for 22% of deaths among children aged 1 to 5 years in 2019 [2] and is also an important cause of death in older adults [3] and immunocompromised people [4]. The burden of pneumonia on health-care resources is enormous due to hospitalization costs and long-term consequences [5]. Infections with bacteria, viruses, fungi, mycoplasma, and chlamydia can all cause pneumonia [6], but the most common pathogeny involves bacterial infections [7].

Presently, antibiotics are used as first-line drugs to treat pneumonia caused by bacteria, mycoplasma, and chlamydia [8]. The incidence and mortality of pneumonia are greatly reduced by the use of antibiotics [9]. However, there are challenges in the antibiotic treatment of pneumonia. The primary threat in the treatment of bacterial pneumonia is the development of antibiotic resistance, which is a global problem [10,11], and it is also an important factor leading to the increasing burden of disease [12]. Compounding the serious problem of antibiotic resistance is a shortage of new antibiotics and the slow pace of drug development [13]. In addition, many challenges exist regarding the usage of antibiotics for the treatment of bacterial pneumonia, including low bioavailability and high side effects associated with traditional strategies, such as oral and systemic administration [14]. While a locoregional delivery of antibiotics into the lung can increase drug bioavailability and decrease systemic side effects, the anatomical, mucous, chemical, and immune barriers that characterize lung tissue hamper the efficacy of antibiotics delivered in this way.

**Figure 1:**
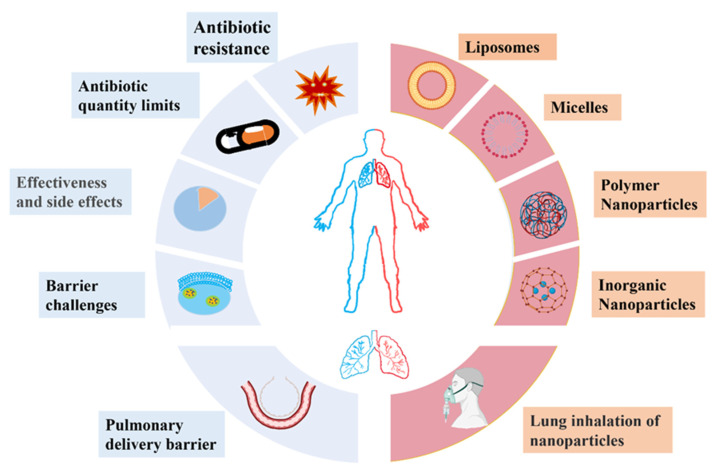
Therapeutic challenges (blue) in the treatment of bacterial pneumonia and novel bioactive nanoparticles (red) that may be applied for the delivery of therapeutic agents.

To address the current limitations of antibiotics in the treatment of pneumonia, new antimicrobial chemicals and mechanisms of antimicrobial action should be developed. The traditional mechanisms of antimicrobial action of antibiotics can be classified into five categories: interfering with cell wall synthesis [15,16], inhibiting protein synthesis [17,18], interfering with DNA synthesis [19], inhibiting bacterial metabolic pathways [20], and damaging bacterial cell membrane structures [14]. In recent years, nanoengineered drug delivery systems have been recognized as a potential new strategy in the fight against bacterial pathogens [21]. The advent of nanotechnology has rekindled interest in the treatment of pneumonia because nano-based drug delivery systems can be used as a tool for the delivery of both systemic and topical therapeutic agents [22,23]. For systemically delivered drugs, nanoparticles can be designed to target the infected region and intelligently release the drugs at the site of infection. This strategy significantly improves the bioavailability of drugs and reduces toxic side effects. For locoregionally administrated nano-based drugs, the pulmonary barriers within the lung can be addressed, as these new drugs show good permeability and are not easily cleared by mucus, but they are readily cleared by lung macrophages (Figure 1).

Nanotechnology helps conventional antibiotics to better reach the target infected area to exert antibacterial activity. The advantage of nanoparticles over conventional antibiotics with a single target of action is their ability to affect the entire bacterial metabolism and other multiple mechanisms of antibacterial activity. Resistance to these nanoparticles re-quires multiple genetic mutations in bacteria, so the probability of bacteria developing nanoparticle resistance through mutation is low. In addition, most of the nanoparticles can be antibacterial through the mechanism of disrupting the bacterial cytosol. The bacterial cell membrane is highly evolutionarily conserved and can hardly be changed by a few genetic mutations, which further reduces the probability of bacterial resistance to nanoparticles. Nanotechnology brings new hope for the clinical treatment of multidrug-resistant bacterial lung infections.

In this review, we present several problems that exist in the treatment of bacterial infectious pneumonia. In addition, in view of the limitations and challenges of current therapies, we highlight the potential of nanotechnology-based delivery systems as a new therapeutic approach. We also describe several nanoparticle types that have been investigated for use in antibiotic delivery and discuss the advantages of inhalation therapy and the obstacles to be overcome in the implementation of this new mode of drug delivery. Finally, we introduce state-of-the-art therapeutic strategies and delivery vectors for the treatment of pneumonia.

## 2. Challenges of Traditional Antibiotic Therapy for Bacterial Pneumonia

**Figure 2:**
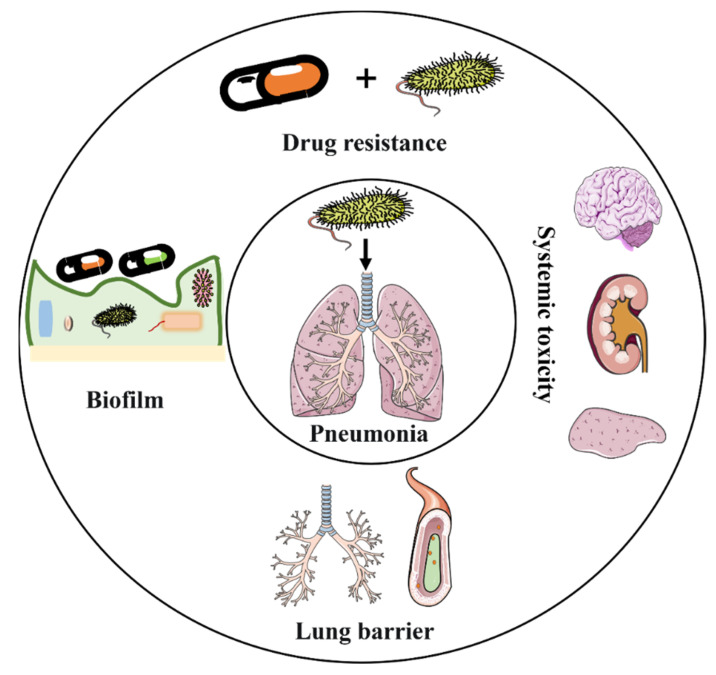
Challenges in the implementation of intravenous antibiotic agents in the antimicrobial treatment of pneumonia.

Antibiotic treatment is associated with multiple important challenges (Figure 2), including (1) increased antibiotic resistance [24,25], (2) limited range of antibiotic agent types [26], (3) low bioavailability [14], (4) adverse side effects [27], and (5) barrier challenges [14].

### 2.1. Antibiotic Resistance

The term “antibiotics” was first used to describe antimicrobial agents in 1941 by Professor Selman Waksman, who discovered more than 20 antibiotics [9]. Since that time, antibiotics have become indispensable in the treatment of various inflammatory disorders [28,29].

**Figure 3:**
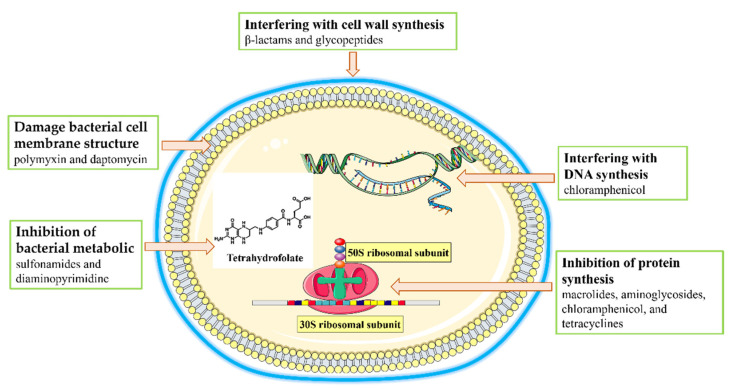
Antimicrobial mechanisms of antibiotics.

There are two main pathways by which antibiotics treat bacterial infections: inhibiting bacterial growth and direct killing of bacteria [13,14]. According to their main principle of action, antibiotics can be divided into five classes (Figure 3). These mechanisms of action include interfering with cell wall synthesis. This class of antibiotics kills bacteria by inhibiting mucopeptide synthetases and hindering the synthesis of the mucopeptides that form the structural basis of the cell wall, causing the swelling and lysis of bacteria [15]. This class of antibiotics includes β-lactams such as penicillin and glycopeptides [15,16]. A second mode of action involves the inhibition of protein synthesis. These antibiotics inhibit the growth of bacteria by binding to the ribosomal subunits responsible for bacterial protein synthesis. For example, the 30S subunit of the bacterial ribosome can be bound by macrolides, aminoglycosides, and tetracyclines to block protein synthesis [17,18]; similarly, chloramphenicol binds to and inhibits the 50S ribosomal subunit [30]. A third type of antibiotic works by interfering with DNA synthesis, such as the inhibition of the replication of bacterial DNA by ciprofloxacin, which alters the superhelix of DNA by binding to bacterial topoisomerases II and IV [19]. A fourth mechanism involves the inhibition of bacterial metabolic pathways. Tetrahydrofolate is required by many bacteria as a one-carbon unit donor [20]; sulfonamides and diaminopyrimidine antibiotics are inhibitors of this pathway [31]. Finally, some antibiotics damage the bacterial cell membrane [14]. For example, positively charged polymyxin and daptomycin interact with and destroy the negatively charged bacterial membrane [32].

**Figure 4:**
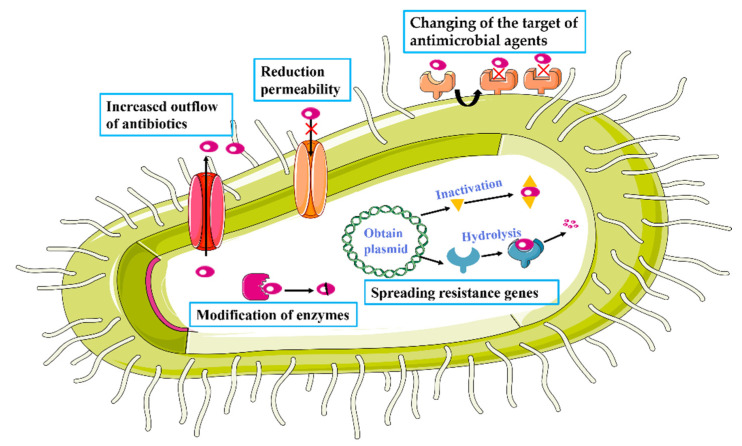
Mechanisms of antibiotic resistance.

In some cases, antibiotics can be rendered ineffective in killing or inhibiting the growth of bacteria through the development of resistance. There are four main mechanisms by which bacteria can exert drug resistance (Figure 4). The first mechanism involves modification by enzymes. Specifically, many antibiotics can be hydrolyzed and neutralized by bacterial enzymes. For example, β-lactamase enzymes disrupt the lactam structure of penicillins and cephalosporins, making them unable to inhibit target mucopeptide synthases [33,34]. Resistance can also be generated by a reduction of permeability or increased efflux of antibiotics. The intake of antibiotics can be reduced by the down-regulation of porin genes or structural modifications or loss of function of the porins that allow antibiotics to enter [13]. On the other hand, efflux of these molecules from bacterial cells can be increased by the up-regulation of active transporters [35]. Resistance can be affected by changes to the antimicrobial target. Accumulation of mutations can lead to the production of target molecules, such as proteins, that are no longer inhibited by the antibiotic. For example, mutation in target enzymes have been shown to cause bacteria to be resistant to quinolones [36]. Once a mechanism is established, it can be broadly disseminated by the spreading of resistance genes. Bacteria can transmit antimicrobial genes or plasmids to naturally susceptible bacteria through transformation [37], transduction [38], and direct contact [39] to facilitate the acquiring of resistance.

In order to overcome the problem of antibiotic resistance, it is important to consider new agents that work in different ways. In addition, the mechanisms of resistance must be considered when applying traditional or novel therapies.

### 2.2. A Limited Range of Antibiotics

Antimicrobial agents commonly used clinically for the treatment of pneumonia include macrolides, amoxicillin, fluoroquinolones, and third-generation cephalosporins [40,41,42]. However, the use of these antibiotics alone or in combination can lead to the emergence of antibiotic-resistant bacteria, making it more difficult to cure infections [24]. Therefore, finding ways to prevent antibiotic resistance from emerging is essential for the treatment of bacterial pneumonia.

The development of new antimicrobial agents is one important strategy to combat resistance. However, despite enormous research efforts and resource consumption, the development of new antibiotics has proceeded slowly [43,44]. In the 1980s, genomics and goal-based screening technologies were adopted by the pharmaceutical industry to accelerate antimicrobial innovation [45,46,47]. Undoubtedly, these technologies for screening drugs were a major advance in the development of new antibiotics [48]. Nonetheless, at present, only 51 new antibiotics have been developed, and only 8 of these drugs can be classified as innovative medicines [26]. What is even more frustrating is the fact that most of these newly developed drugs simply involve the chemical modification of existing drugs [46]. The development of new antimicrobial agents is slowed by several factors: the research is time consuming [49], the high investments yield at best low profits [50,51], and a long time is required to obtain approval from drug regulators.

### 2.3. Low Bioavailability and High Side Effects of Antibiotics

The administration of antibiotic drugs usually occurs via oral or intravenous routes, which result in systemic distribution. Only a small amount of the drug reaches the site of infection [14]. For example, after oral administration of fluoroquinolones, much of the drug is rapidly excreted through the biliary system, and approximately one-third of the drug is eventually present in the stool [52]. In order to ensure a therapeutic effect, it is often necessary to administer large doses and treat for long periods, which can lead to undesired toxic side effects and drug resistance [14]. Alternatively, if large doses are not feasible, the infectious bacteria are exposed to sub-inhibitory concentrations of drugs, whereupon they become more susceptible to adaptive mutations and other genetic changes, leading to an increased risk of drug resistance [53]. 

The adverse side effects caused by the large doses also hamper the use of antibiotics in clinical treatment. For example, high doses of nitrofurantoin may lead to pulmonary toxicity [27], linezolid may lead to hematologic toxicity [27], metronidazole may cause neurotoxicity [27], fluoroquinolones may increase the risk of aortic aneurysm [27], and gentamicin is often associated with acute kidney injury [54].

In addition to direct injuries to the patient, the gut microbiota, which is composed of a variety of commensal bacteria that resist colonization and invasion by pathogens, will also be disturbed by large-dose oral or systemic antibiotics. Antibiotic exposure can lead to an imbalance in gut bacteria, which increases susceptibility to infection [55] and which is also linked to a number of noncommunicable diseases [56]. Damage to the gut microbiota by antibiotic exposure can be long-lasting; for example, long-term disturbances in microbiota compositions have been observed even after short-term clindamycin exposure [57].

### 2.4. Barrier Challenges in Pneumonia Treatment

The treatment of pneumonia faces challenges posed by multiple types of barriers. These barrier-related challenges can be divided into different categories according to the routes of administration of the antibiotics.

#### 2.4.1. Barrier Challenges for the Systemic Delivery of Antibiotics

**Figure 5:**
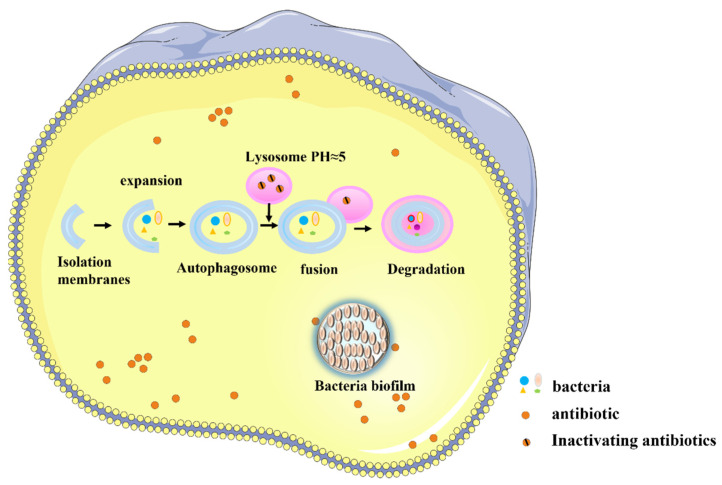
Challenges in the systemic delivery of antibiotics.

Systemic antibiotic delivery faces multiple obstacles and barrier-related challenges (Figure 5). In some cases, the host cell membrane represents a barrier. Some bacteria, such as *Legionella pneumophila* [14], enter the cytoplasm of host cells, lowering the killing effect of antibiotics [58]. In this case, the cell membrane barrier causes several challenges. The antibiotics that are applied to kill intracellular bacteria must reach adequate concentrations of active drugs in cells, and they must be retained for a sufficient time. Those antibiotics that do not readily enter cells, such as β-lactam drugs, have been shown to achieve intracellular concentrations that may be too small to be effective. Once in the cell, the drug molecule will be subject to active export, often leading to half-lives that are too short [59].

Barriers may also be presented by organelles and organellar membranes. Host cells may form phagosomes via the phagophore in order to recognize and bind to bacteria as a defense mechanism. These structures bind to lysosomes to form autolysosomes that may kill the bacteria [60]. However, some bacteria have evolved immune escape mechanisms that can inhibit the bactericidal effects of autolysosomes [61]; these bacteria can survive and even reproduce in phagosomes and phagolysosomes [62]. This bacterial defense mechanism further protects these bacteria from antibiotics, as some antimicrobial compounds do not readily enter the organelles. For example, clarithromycin has been shown to be excluded by the phagosome where bacteria often reside [63]. Moreover, even if the antibiotic enters the organelle, its activity may be impaired in the acidic environment of the lysosome [59].

A third type of barrier is presented by the formation of a bacteria biofilm barrier. Some bacteria can form protective biofilms [64], which increase the speed with which antibiotic resistance is acquired by bacteria by up to 1000-fold [65]. The biofilm itself also presents a physical barrier that directly hampers the efficacy of antibiotics [66,67]. All of these barriers make it difficult for systemically delivered antibiotics to contact bacteria, thus lowering the inhibitory or killing potency.

#### 2.4.2. Barrier Challenges for the Local Delivery of Antibiotics

Local delivery of antibiotics can circumvent first-pass metabolism, minimize systemic side effects, decrease inactivation by metabolic enzymes, enhance drug bioavailability, and facilitate the initiation of therapeutic action [68,69,70]. Nevertheless, in the case of antibiotics used to treat bacterial pneumonia, local delivery of the drug to the lungs through the respiratory tract also faces multiple obstacles. In addition to the barrier challenges presented by cells, organelles, and bacterial biofilms, local drug delivery within the lung must overcome the biological barriers presented by structures and functions specific to the respiratory tract.

The lungs exhibit strong innate defenses and physical barriers that make it difficult for inhalable drugs to reach the site of action. When inhalable drugs do reach deep within the lungs, they are rapidly cleared or inactivated by biological defense mechanisms. Therefore, drug delivery to pulmonary targets is not an easy task.

**Figure 6:**
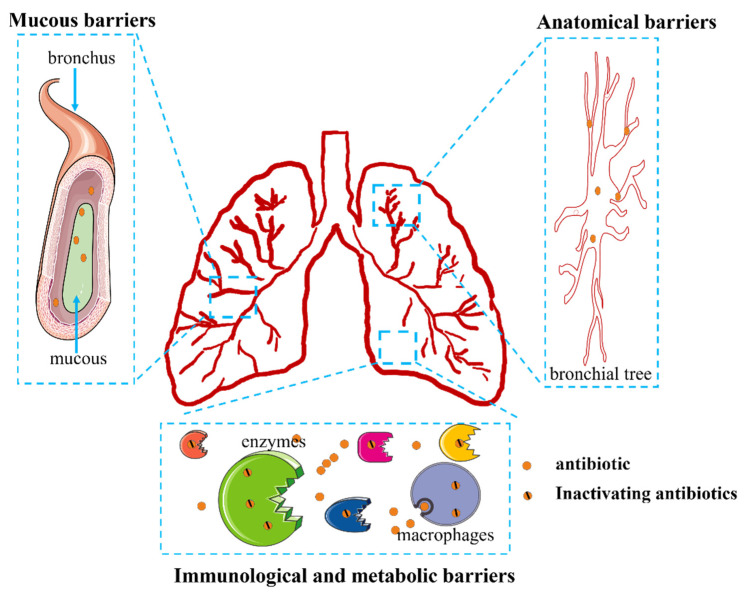
Challenges in the local delivery of antibiotics.

Targeted delivery to the lungs requires overcoming three main known barriers. One type of barrier is known as an anatomical barrier. The lungs have a complex bronchial tree lined with ciliary cells that exert pulmonary mucociliary clearance to remove particles deposited in the airways. These functions create a mechanical barrier that inhibits pulmonary drug delivery [71]. A second type of barrier involves the mucous barrier. Respiratory mucus covering the airways from the nose to the fine bronchial tubes allows the capture and removal of foreign bodies, including xenobiotic compounds such as antibiotic agents [71]. In addition, the mucous barrier works synergistically with anatomical barriers, and lung mechanical barriers become more effective due to airway stenosis caused by inflammation and mucus hypersecretion [71]. Immunological and metabolic barriers also influence drug delivery. Since the function of the lungs is to participate in the exchange of air with the outside world, they face a complex external environment; therefore, the lung forms a strong barrier consisting of chemical and immune defenses. The chemical and immune barriers to drug delivery in the lungs consist of proteolytic enzymes, surfactants, and alveolar macrophages [72]. Proteolytic enzymes, including endopeptidase and cathepsin H, can hydrolyze and thus inactivate drugs [72]. Many macrophages are also present in the alveoli, and alveolar phagocytes can engulf and remove particles that reach the alveoli. Chemical surfactants augment these functions by preventing inhaled particles from adhering to the epithelial surface of the lung and facilitating their removal by macrophages [73,74]. It is clear, then, that effective pulmonary inhalation drug delivery requires overcoming formidable barriers (Figure 6).

## 3. Bioactive Nanoparticles for the Treatment of Bacterial Infections in the Lungs

In current practice and in emerging clinical trials, most pulmonary infection treatments are administered through oral and intravenous routes, despite the fact that most antibiotics and anti-inflammatory drugs are known to be eliminated rapidly from the circulatory system [75]. Without a mechanism to specifically target drugs to the lungs, drugs are distributed passively in the body, leading to low effective drug concentrations at the site of infection. These low concentrations reduce the therapeutic effect and are conducive to the development of drug resistance [76].

The delivery of antibiotics by nano-based drug delivery systems represents a promising way to address these challenges. Nanodelivery systems can be engineered to change the intrinsic physical and chemical properties of conventional antibiotics and provide characteristics that preferentially target them to the appropriate site. More recently, smart nano-based drug delivery systems have been designed to precisely target the desired region and responsively release the drugs under specific stimuli; these systems have been demonstrated to enhance local drug concentrations and to reduce side effects in healthy areas [77]. In this regard, there have been multiple studies focusing on the microenvironment of lung infection. When bacteria infect the lung, a series of physiological index changes such as inflammation, lowered pH, and increased levels of certain enzymes and reactive oxygen species (ROS) occur at the infection site [78]. All of these abnormal indexes can serve as stimuli for the smart nanosystems, enabling the directed release of drugs.

The application of nanocarriers to address bacterial infections in the lung has become a common tool. Antimicrobial drugs are delivered to the bacteria in the lungs in order to exert their antimicrobial effect. The inability to completely eradicate the bacteria from the infected area is a consequence of the development of drug resistance [79]. After the drug is injected intravenously into the organism and circulated throughout the body to the organs, the first problem is how to enhance the process of accumulation of the drug in the lungs [80]. The size of the particles greatly affects the pulmonary targeting ability of the drug. For systemic circulation, the drug dose needs to be increased to maintain the therapeutic effect, which to some extent leads to bacterial resistance. Modification of nanocarriers and control of nanoparticle size can confer active lung targeting properties to the drug [81]. The ability of the materials in question to target the infected microenvironment of the lung further addresses the problem of antimicrobial drug resistance. Drugs are released upon reaching the infected environment of the lung and are unable to interact with bacteria due to the cell membrane or biofilm barrier. Using nanocarriers with the ability to penetrate cell membranes can deliver antimicrobial drugs into the cells and address the problem of intracellular infections [82]. Some studies have reported that the use of guanidinylated polycarbonate materials as adjuvants can help antimicrobial drugs reach intracellularly. Even after incubation with bacteria for tens of generations, there was no problem of bacterial drug resistance. The presence of biofilm hinders drug penetration, resulting in poor therapeutic efficacy. Nanomaterials increase the ability of drug penetration and penetration in biofilms to reach deep into the biofilm and bind to bacteria. The specific surface structures of some materials anchor to the bacterial surface and exert synergistic antibacterial activity. Helping drugs bind to bacteria by overcoming lung-associated barriers is the key to nanotechnology for treating pneumonia bacteria [83].

**Figure 7:**
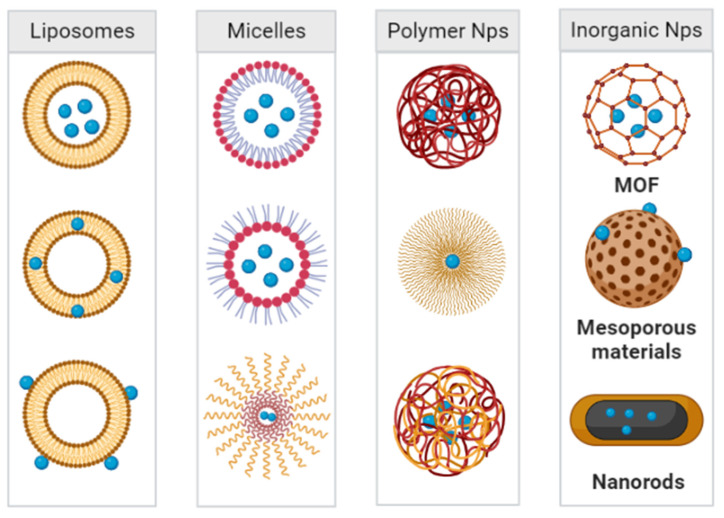
Nanoparticle systems currently under study for the delivery of antibacterial agents in the treatment of pneumonia.

Various bioactive nanoparticles have shown potential as carriers for pneumonia treatments. The timing and concentration of therapeutic drug accumulation at the site of infection are critical to the effective treatment of pneumonia [84]. Among many bioactive nanoparticle systems applied for the treatment of bacterial lung infections, polymeric nanoparticles are one of the most commonly used nanoagents. For example, nanoparticles based on poly (lactic-*co*-glycolic acid) (PLGA) have been employed to effectively treat pneumonia, and their key advantages lie in their non-toxicity and biodegradability. In addition, polyethylene glycol (PEG) is another commonly used material for carriers for lung drug delivery systems [85]. To this point, several such materials have been approved by the Food and Drug Administration (FDA) as carriers for drug delivery systems, including PLGA and chitosan. In addition to polymer nanoparticles, liposomes, micelles, and inorganic nanoparticles (e.g., gold nanoparticles, iron nanoparticles, and quantum dots) have also been applied as antibiotics carriers. These particles have large areas that can be modified with functional moieties, including targeting molecules and environmentally responsive moieties (Figure 7). Research regarding these and other micellar, inorganic, and metallic materials is currently identifying more effective nanomaterials for antimicrobial therapy in the lung [86].

Despite the promise of nanocarriers in pulmonary medicine, most drug carriers are foreign substances. We need to consider the potential risks due to interactions between nanomaterials and biological systems. Ideally, nanomaterials should perform functions in the lungs and then be excreted from the body without any harmful effects [87]. However, the introduction of foreign molecules may significantly alter physiological responses and affect pharmacokinetics, an field that lacks laboratory studies. In addition to the interaction of nanocarriers with organisms, the interaction of nanocarriers with bacteria must be explored by further studies. The effects of long-term use of nanocarriers on bacterial structure and genetics, among others, are not yet known. From the clinical point of view, there are still many problems to achieve large-scale preparation of nanomedicines. Firstly, the cost of raw materials has to be considered; some polymers are expensive to synthesize and purify, such as PLGA-b-PEG and PEG-b-PLA [88]. Secondly, poor stability of drug-material binding and low drug-loading capacity hinder the transition of nanomedicines to clinical therapeutics. How to achieve the controlled preparation of nanomedicines and balancing the therapeutic effect of drugs with the cost of raw materials are future challenges. In addition, clarifying the metabolic processes of nanocarriers in the lung and in vivo are also issues that need attention [89].

### 3.1. Liposomes

Liposomal delivery of antibiotics for the treatment of pneumonia is currently being explored. The components of liposomes, especially phospholipids, are biocompatible and biodegradable. Liposomes can act as drug carriers, increase the concentration of drugs in the body, protect encapsulated drugs, and allow the controllable release of drugs [90,91]. Delivery of antibiotics with liposomes increases cellular uptake, effectively reducing the dose of the drug and decreasing toxicity. Transpulmonary delivery of drug-carrying liposomes can also reduce drug toxicity to lung tissue by preventing local irritation. By promoting uptake by tracheal epithelial cells and alveolar epithelial cells, the compounds reach the circulation intact, thus enhancing the therapeutic effect of the drug [92].

The enhanced uptake of liposomes into cells can facilitate the delivery of antimicrobial drugs to intracellular compartments. For example, Su et al. designed a multifunctional copolymer en route to the development of polymer-enhanced liposomes (PALs). These PALs lead to a specific targeting of alveolar macrophages via a mannose moiety, and they were found to improve the efficiency of delivery of streptomycin to the target cells [93]. In another work, Rathnayake et al. designed lipid-encapsulated targeted nanoassemblies to deliver antibiotics to the environment of bacteria or even to the bacterial cytoplasm. The liposome shell prevented premature drug release and degraded only after exposure to lipase secreted by *Pseudomonas aeruginosa*, successfully targeting and inhibiting the growth of *P. aeruginosa* in lung epithelial cells [94]. Lung-targeted nanovesicles composed of lipids also have been shown to eliminate both extracellular and intracellular drug-resistant bacteria, potentially addressing the problem of bacterial drug resistance [95].

**Figure 8:**
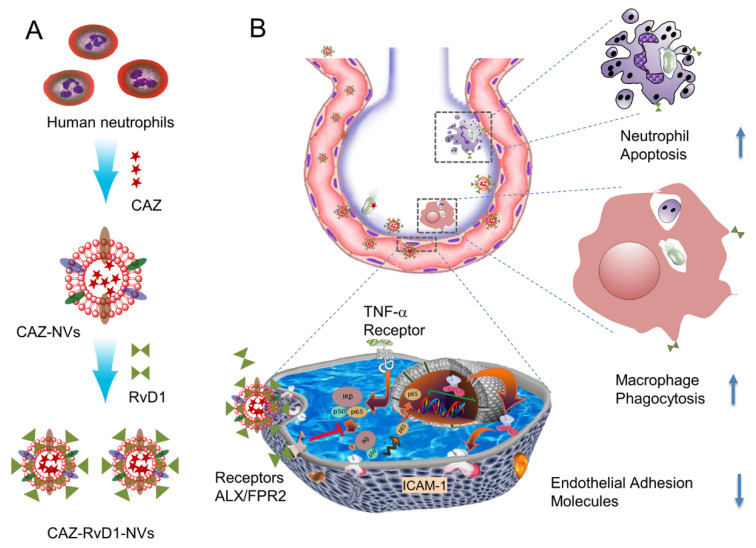
(**A**) shows the generation of neutrophil membrane-derived nanovesicles (NVs) co-loaded with RvD1 and Ceftazidime (CAZ). (**B**) The nanovesicles home to the infectious lung deliver both RvD1 and CAZ. RvD1 binds to GPCRs (ALX/FPR2), inhibiting the NF-κB pathway to decrease the expression of cell adhesion molecules, and mitigating neutrophil tissue infiltration and increasing the phagocytosis. Simultaneously, CAZ inhibits bacterial growth in the lung. Modified and reproduced with permission from the Spring [98].

In addition to the elimination of bacteria from the lungs, treatment of inflammation caused by bacterial infections is also a matter of concern. Recently, Allemailem et al. prepared thymoquinone-based liposomes and found that they exhibited both antibacterial and antibiofilm activity in the treatment of *Acinetobacter baumannii* infections [96]. Drug co-delivery with liposome systems may be an effective way to address both inflammation and infection [97]. In one study, the anti-inflammatory agent resolving D1 was combined with the antibiotic ceftazidime in a nanoagent to target an inflamed vascular system (Figure 8). After administration of this combination to mice, cytokine levels were found to be reduced and bacterial growth in the lungs was also inhibited [98].

To address the effect of biofilm formation on drug concentrations at sites of infection, Rao et al. proposed an antibiotic adjuvant liposome with a negatively charged surface. This hydrophilic particle readily penetrated the sputum layer, with its degradation and drug release facilitated by phospholipase A2 accumulated in the microenvironment of a *P. aeruginosa* biofilm. The liposomes enhanced the antibacterial activity of azithromycin, which had a significant effect on *P. aeruginosa* and prevented the bacteria from adhering to airway epithelial cells, thereby preventing recurrent infections [99].

In an ideal drug therapy system, the drug should only be released at the site of bacterial infection. Fortunately, liposomes can be modified with chemical moieties that endow them with a pneumonia-targeting ability. For instance, Pushparaj et al. integrated a siderophore, pyochelin, into liposomes. This targeted nanoparticle was shown to have strong activity against *P. aeruginosa* and was able to limit the toxicity of the drug [100]. Antibiotics loaded into the liposome core can take advantage of specific transmembrane pores induced by the liposomes and can be released at the site of infection, augmenting the effectiveness of the therapeutic agents [101].

Several inhalable liposomal formulations have been used in the clinic to deliver drugs directly to infected pulmonary cells, thereby speeding the onset of treatment effects. Recently, mannose-modified liposomes have been designed as inhalable formulations for the treatment of latent tuberculosis infections. These nanoparticles, which also have macrophage targeting and pH-sensitive characteristics, were shown to eliminate *Mycobacterium tuberculosis* from macrophages [102]. The uniform encapsulation of drugs in liposomal particles by spray-drying techniques can also protect the compounds from degradation by biological or chemical barriers [103].

### 3.2. Micelles

Many studies have focused on the application of micelles in pneumonia therapy. Since micelles are self-assembled from amphiphilic copolymers and they exhibit some advantages over other types of carrier materials. For example, they tend to have higher stability, stimuli-responsive properties, and higher biocompatibility than other particles, and they can be used to load hydrophobic drugs [104]. Many indicators within the bacterial infection microenvironment, such as altered pH and changes to levels of reactive oxygen species and enzymes, can serve as specific targets and responsive stimuli for micellar nanoparticles [105]. In addition, micelles are capable of self-assembling around hydrophobic drugs to achieve targeted and responsive release for drug design needs. In a recent study, Chen et al. loaded vancomycin into nanonuclei, which effectively reduced the bacterial burden and alveolar damage in the lung. After reaching the site of infection, these micellar particles released the antibiotics due to bond lysis occurring in the more acidic pH environment of the infection, and the normal alveolar microstructure was achieved [106].

**Figure 9:**
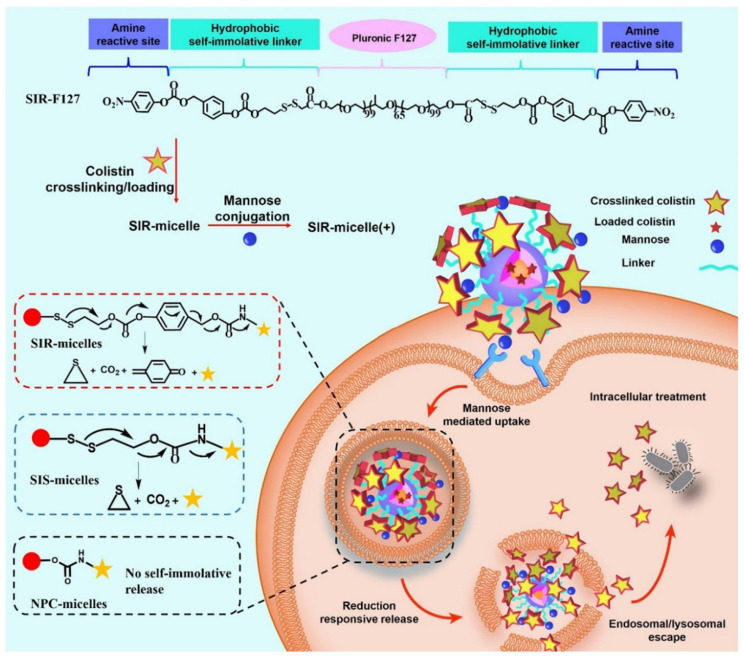
Schematic illustration of the treatment of intracellular bacterial infections using SIR-micelles(+). SIR-F127 has three moieties: pristine F127 used for conjugation scaffold and micelle formation; hydrophobic and self-immolative moiety used for cell penetration and traceless drug release; amine active moiety used for colistin or mannose crosslinking and conjugation. SIR-micelles(+) can be easily generated by adding colistin and mannose successively in SIR-F127 aqueous solution. Modified and reproduced with permission from the Elsevier [107].

Micellar nanoparticles have achieved good efficacy in the removal of intracellular bacteria, the eradication of bacterial biofilms, and the treatment of inflammation. For example, Yang et al. designed micellar particles to enhance drug accumulation in macrophages by modifying the micellar surface with mannose (Figure 9). These nanoparticles rapidly released antibiotics upon the cleavage of disulfide bonds in the reducing intracellular environment, thus achieving targeted treatment of intracellular drug-resistant pathogens [107]. Other targeted smart micelles have been shown to selectively bind with different types of bacterial surface receptors, achieving the precise release of drugs [108,109].

Micellar materials also have the potential to be applied to bacterial imaging. In particular, they show great potential as targeted imaging and treatment agents in the detection and elimination of infectious bacteria. Park et al. reported a new family of antimicrobial agents that are constructed by self-assembly of chimeric antimicrobial lipopeptides and other polymers. The antimicrobial lipopeptide consists of distearoyl phosphatidylethanolamine (DSPE) and HnMc linkages. HnMc micelles are highly targeted to the site of bacterial infection and are effective in killing drug-resistant bacteria. Such an antimicrobial lipopeptide and amphiphilic copolymer design strategy will be useful in the detection and treatment of bacterial infections [110].

Similarly, micelles have been applied to the treatment of sepsis. A combination of antibiotics and anti-inflammatory drugs in the micelles stops the spread of bacteria and relieves the inflammation caused by bacteria. Thus, the systemic bacteremia and excessive inflammation that cause sepsis are avoided [111]. Similarly, Zhang et al. synthesized micellar nanoparticles through the self-assembly of 1,2-distearoyl-sn-glycero-3-phosphoethanolamine-poly(ethylene glycol) with appropriate drugs. This assembly formed copolymers that are sensitive to pH and bacterial enzymes and thus are responsive to the infection microenvironment. Application of this system to a mouse model of sepsis led to the reduction of systemic bacteria, white blood cells, and inflammatory cytokines [112].

### 3.3. Polymer Nanoparticles

Polymeric nanoparticles have been widely applied in studies of lung infections. There are multiple types of polymers that can associate with different drug molecules and that are biocompatible, highly functional, and exhibit low toxicity. Recent studies have shown that nanoparticles consisting of polymer-loaded antibiotics greatly reduce the toxicity of the drug but retain antibacterial activity. These nanoparticles have especially been applied to the reintroduction of older antibiotics into the clinic. For example, polymyxin B (PMB) is effective against multidrug-resistant Gram-negative bacteria, but their nephrotoxicity and neurotoxicity limit the doses that can be administered. However, a recent study using polymer-delivered mucin showed good efficacy [113]. Here, the authors modified PMB with 2,3-dimethylmaleic anhydride and grafted oligomeric chitosan to prepare polyionic nanocomplexes. The polymers shielded the positive charge of PMB by electrostatic interactions, reducing the side effects of the drug but retaining its antimicrobial activity.

**Figure 10:**
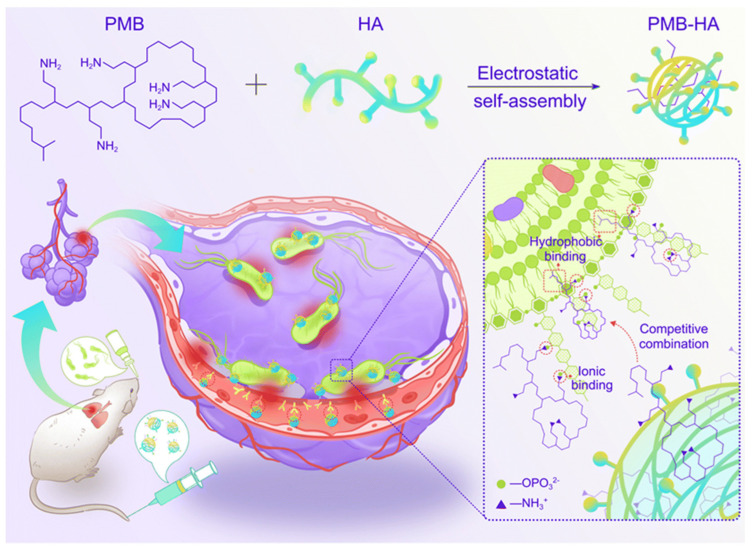
Schematic illustration of the construction of inflammation-targeted smart nanoparticles PMB-HA, together with the molecular mechanism of their bacteria-triggered PMB-forced-release ability to precisely kill the pneumonia bacteria with outstanding biosafety. Modified and reproduced with permission from the Royal Society of Chemistry [115].

Other similar studies have shown that electrostatic assembly of polymyxins with polyanionic materials leads to good results. The targeted-release properties of the drug imparted by the polymeric material could be a general approach to improve other highly toxic antibiotics [114]. Zhang et al. produced polymer nanoparticles through self-assembly of negatively charged hyaluronic acid (HA) with positively charged PMB molecules (Figure 10). After intravenous injection, the nanoparticles were found to aggregate in the infected area of the lung due to the targeting of HA to CD44 receptors overexpressed on endothelial cells in the inflammatory state. Due to the antibacterial activity of PMB in which it binds to the phosphate group of lipopolysaccharides (LPS) in the bacterial outer membrane, the drug in PMB-HA nanoparticles is competitively released upon the encountering of bacteria. Furthermore, by reducing the positive charge of PMB, PMB-HA nanoparticles reduce cellular damage as well as histological toxicity, thus improving cellular and biological safety. This intelligent delivery system provides a new approach for the resurrection of PMB in the treatment of bacterial inflammatory diseases [115].

Polymers can be used both as drug carriers and in combination with other therapeutic modalities to exert combined therapeutic effects. In addition to delivering antibiotics, polymers combined with antibodies, vaccines, and phages can have a dual therapeutic effect [116,117,118,119]. For example, Hussain et al. combined vancomycin-loaded nanoparticles with phage-recognized amino acid peptides, and the combined delivery of the two drugs improved the antimicrobial activity of the nanoparticles in vivo. This approach reduced the systemic dose of the drug required and minimized side effects [120]. Overall, polymeric materials offer drug delivery strategies for the synergistic treatment of bacterial resistant infections.

### 3.4. Inorganic Nanoparticles

The threat of antibiotic resistance has led to an urgent need to develop new antibacterial compounds, with inorganic nanoparticles being used as antibiotic alternatives. Related inorganic antimicrobial materials have shown good antibiotic-like activity. The synergistic antibacterial activity of inorganic antimicrobial materials with antibiotics can help achieve better therapeutic results. Recently, researchers reported a material that has not led to drug resistance, even with repeated use. The material is biodegradable and has achieved positive therapeutic results for a wide range of bacterial infections [121]. The related synthesized compounds have been shown to have antibacterial activity against most bacteria, with the antibacterial activity further improved by structural modifications [122,123,124].

In addition to biodegradable antimicrobial polycarbonates, other antimicrobial materials have been reported to address drug resistance as well as toxicity issues and have good potential for clinical applications. Some inorganic antimicrobial materials, such as nanorods and nanosheets, exhibit the potential to enhance the accumulation of drugs in bacteria. Accordingly, related antimicrobial material delivery systems show improved results in treating bacterial infections [125,126]. In addition to the aforementioned inorganic nanoparticles, metallic nanomaterials with tunable size have also been applied as nanodrug delivery materials. For example, the highly porous zeolite–imidazolium salt backbone (ZIF-8) offers tremendous advantages in the construction of drug delivery systems when it is compounded with HA. HA targets CD44 receptors on macrophages leading to cellular uptake and the intracellular delivery of associated antibiotics. In the acidic environment of lysosomes in macrophages, the material structure can be disintegrated to release the drug, which can then eradicate intracellular pathogenic bacteria [127]. These results suggest that metallic nanomaterials will be useful as an alternative antibacterial delivery system [128].

**Figure 11:**
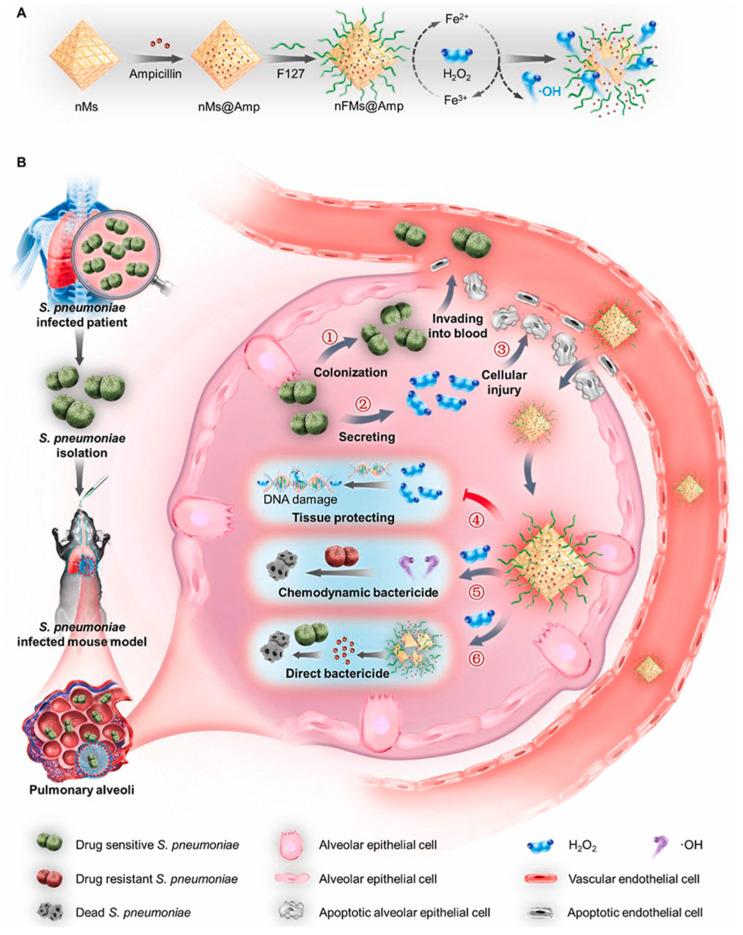
Schematic illustration showing the working principles of nFMs@Amp to treat hydrogen peroxide (H_2_O_2_)-secreting *Streptococcus pneumoniae* (*S. pneumoniae*) infection. (**A**) Schematic diagram of the synthesis steps of nFMs@Amp. (**B**) Schematic diagram of the process of *Streptococcus pneumoniae* causing lung infection in mice and the mechanism of action of nFMs@Amp in eliminating H_2_O_2_ from the lungs of infected mice. Modified and reproduced with permission from the Elsevier [129].

By diminishing the inflammation caused by bacterial infection, relevant nanometallic compounds or enzymes can reduce pneumonia-related damage by eliminating excess reactive oxygen species in the inflammatory environment. Wu et al. proposed to deplete H_2_O_2_ from bacterially infected lungs by reacting H_2_O_2_ with MOFs to protect tissue and prevent sepsis (Figure 11). Inorganic nanoparticles were prepared by loading Fe^3+^-doped MOFs with ampicillin (nFMs@Amp). MOFs effectively accumulated in the lungs after systemic administration due to infection-induced alveolar–capillary barrier dysfunction. In addition, the nanosystem has a good chemical kinetic bactericidal effect on drug-resistant bacteria. By synergistic treatment with the antibiotic ampicillin, MOFs eliminated more than 98% of invading *Streptococcus pneumoniae* [129]. For example, related work on the removal of H_2_O_2_ opens new avenues for the clinical treatment of toxin-secreting bacteria. Here, a nanoenzyme was found to be effective in reducing the concentration of H_2_O_2_ that was stimulated by the presence of a bacterial infection. The reduction of H_2_O_2_ concentrations to physiological levels also facilitates the breakdown of biofilms in vivo and the prevention of new biofilm formation, which would be expected to accelerate the repair of tissue damage [130]. Combining such materials with photothermal therapy would enhance the addressing of the problem of biofilm resistance. This strategy thus provides a promising strategy for non-antibiotic therapy [131,132].

## 4. Pulmonary Inhalation of Nanoparticles in the Treatment of Pneumonia

**Figure 12:**
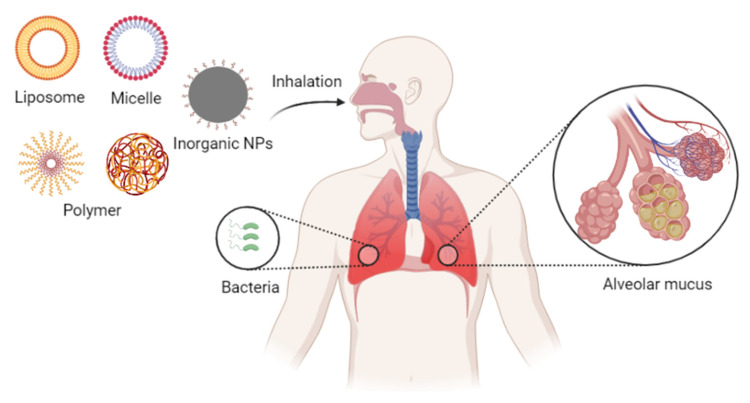
Inhalation nanoformulations for overcoming pulmonary delivery barriers.

Both the local concentration and the retention time of the drugs within an inflammatory lesion can impact clinical outcomes. Delivering the drug directly to infected cells through the alveoli and bronchi can shorten the time to onset of action compared to the systemic delivery route. Therefore, inhaled drugs are gradually gaining attention among researchers [133,134,135,136]. Systemic administration requires specific targeting to achieve an optimal effect, but local delivery to the lungs provides more direct targeting. Pulmonary inhalation administration can significantly increase local drug concentrations, thereby reducing the necessary dose. In other words, local delivery can enhance the drug’s effectiveness. Inhaled drug delivery thus remains a preferred clinical approach for the treatment of various lung-related diseases, including acute lung injury and asthma. Pulmonary delivery of nebulized drugs also offers a potential way to treat bacterial infections of the lung (Figure 12).

### 4.1. Pulmonary Drug Delivery Barriers

Effective pulmonary inhalation drug delivery requires overcoming relevant barriers, including anatomical, physical, immunological, and metabolic barriers. Due to the physiological limitations of the lung, it is reported that inhaled formulations with particle sizes smaller than 1 to 3 μm are required for deep delivery into the alveoli. Therefore, nebulizing of the nanodrug formulations to the appropriate formulation size is necessary [137,138]. With respected to the physical barrier, respiratory mucus covers the airways from the nose to the fine bronchial tubes for the capture and removal of foreign bodies. Respiratory secretions in the airways and alveolar linings may also trap polymer particles or reduce their stability. The mucus screens out nanoparticles larger than the grid spacing of the mucin network, which is approximately 100 to 400 nm. In addition, mucus also slows the diffusion of molecules smaller than the sieve spacing, constructing a dynamic barrier for the protection of the physiological state of the lung.

In addition to the mucosal barrier, nanoparticles may be cleared by macrophages and epithelial endocytosis in the alveoli [139]. Nanoparticles of 260 nm and smaller have been reported to be readily transferred from the lung into the systemic circulation, which is detrimental to the efficacy of locally delivered drugs, and it may increase systemic side effects. Due to the limitations of the pulmonary drug delivery barrier, specific requirements are placed on the nature of the drug delivery system. Firstly, the inhaled formulation should be nebulized into droplets of appropriate size for effective deposition into the alveoli. Secondly, hydrophilic and electroneutral nanoparticles are preferred for their propensity to penetrate the mucus barrier and successfully reach the cell surface. Finally, to meet metabolic requirements, the carrier system also needs to be biodegradable and neither cytotoxic nor immunogenic. This requires carrier materials with suitable size and surface properties to achieve higher delivery efficiency, better drug efficacy, and optimal safety profiles.

### 4.2. Nanoparticles for Pulmonary Inhalation

Direct delivery of antibiotics via pulmonary inhalation has been practiced in the clinic. For example, while antimicrobial peptides (AMP) are effective therapeutic agents, traditional systemic routes of administration may lead to undesired effects, including low bioavailability and high toxicity. In this context, the pulmonary inhalation route has been found to address these limitations. A study by van der Weide et al. found that novel antimicrobial peptide nanomedicines (AA139) were therapeutically effective in the treatment of sepsis caused by multidrug-resistant *Klebsiella pneumoniae* when they were encapsulated into nanoparticles by polymers or micelles. The therapeutic effect of nanoparticles containing AA139 on rats with pneumonia-related sepsis was measured after tracheal intubation aerosolization. These nanodrugs significantly prolonged survival time and thus acted as an effective treatment of pneumonia-related sepsis caused by multidrug-resistant Gram-negative bacteria [140]. Non-natural antimicrobial peptides have also been reported to show in vitro efficacy against a wide range of drug-resistant strains and to resist degradation in biological fluids [141]. The development of multifunctional antimicrobial peptides and the preparation of biodegradable nanoparticles have expanded the range of applications of antimicrobial drugs [142].

**Figure 13:**
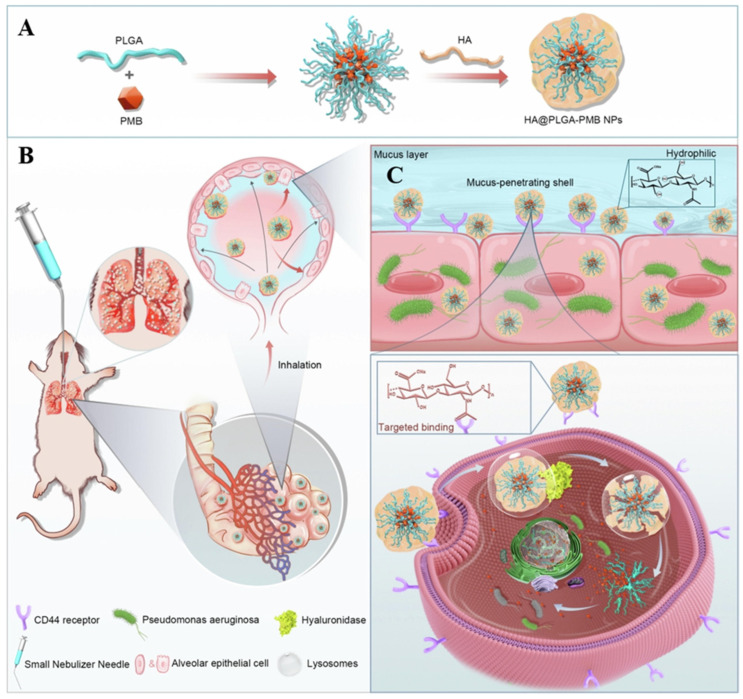
Schematic diagram of the preparation and nebulization of HA@PLGA-PMB nanoparticles for the treatment of lung infections. (**A**) Synthesis route of HA@PLGA-PMB nanoparticles. (**B**) Schematic diagram of nebulized administration of HA@PLGA-PMB nanoparticles in a mouse lung infection model. (**C**) Mucus penetration and cellular uptake processes of HA@PLGA-PMB nanoparticles. Modified and reproduced with permission from the Elsevier [145].

The pulmonary barrier limits the scope of antibiotic applications for pulmonary inhalation delivery. Most antibiotics cannot be delivered directly to the lungs due to their inherent limitations, such as high hydrophobicity and poor cellular absorption [143]. The introduction of nanomaterials has greatly improved the efficiency of pulmonary drug delivery and significantly reduced drug toxicity. Among the common lung delivery vehicles, PLGA-related polymers have been used to carry several types of drugs, such as proteins, peptides, DNA, and siRNA. Casciaro et al. delivered PLGA-encapsulated antimicrobial peptides via a liquid injector to a mouse model of pneumonia. The PLGA nanoparticles inhibited bacterial growth, leading to a reduction in lung bacterial load [144]. PLGA nanoparticles have also been used to aid drug penetration through the mucus barrier and improve the efficiency of pulmonary drug delivery. Wu et al. constructed nanoparticles of hydrophilic HA with PLGA containing PMB to enable better penetration of the drug through the mucus barrier (Figure 13). The modification of PLGA improved the drug delivery efficiency and promoted the slow release of the drug for sustained treatment [145].

In addition, polymeric PEGs have also been widely used as nanomaterials for pulmonary drug delivery by inhalation. Hydrophilic and electroneutral PEGs have shown promise in overcoming the mucus barrier for better drug delivery to infected cells. Drug PEGylation imparts a hydrophilic layer to the drug, changing its hydrophilicity and size to permit its penetration through the mucus layer [146]. In addition to the delivery of traditional antimicrobial drugs, PEG is also commonly used for delivery of siRNA for anti-inflammatory therapy. This nanocomplex with dual permeability to both mucus and cell membranes promises to become a practical tool for the treatment of severe pneumonia [147].

**Figure 14:**
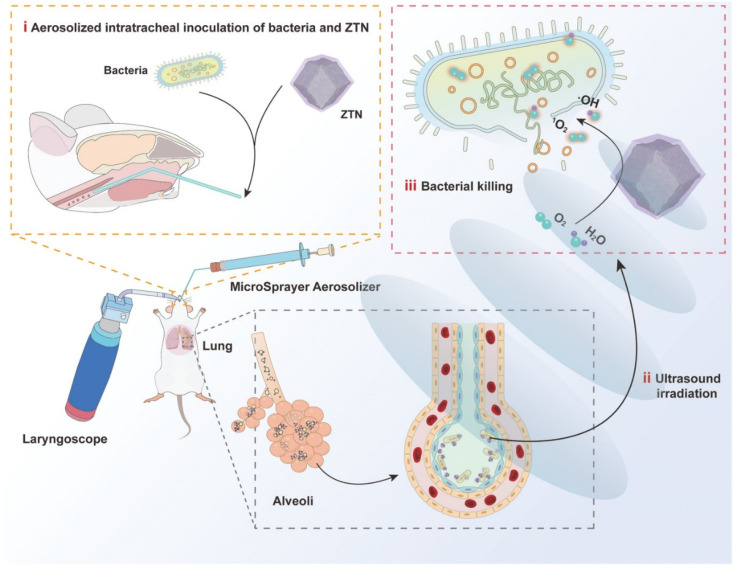
Schematic illustration of ZTNs for SDT of bacterial lung infections. Modified and reproduced with permission from the Wiley-VCH GmbH [148].

With the development of nanotechnology, inhalation therapy combined with various treatments, such as phototherapy and sonodynamic therapy (SDT), has been found to effectively remove bacteria and avoid antibiotic resistance. Due to the strong tissue penetration of ultrasound, SDT has potential applications in the treatment of deep tissue diseases. The use of ultrasound to activate acoustic sensitizers to produce different reactive oxygen species leads to a strong antibacterial effect. In a recent study, metal-organic framework-derived nanoparticles were designed to be used in inhaled treatments of bacterial pneumonia. ZIF-8 was incorporated into these nanoparticles as an acoustic sensitizer to generate bactericidal reactive oxygen species upon sonodynamic therapy [148]. Similarly, upon nebulized intratracheal inoculation, nanoparticles constructed of ZrTi_2_O_6_ have been found to aggregate in the infected areas of the lung. In addition, after ultrasound irradiation in vitro, this material produces reactive oxygen species that kill Gram-negative multidrug-resistant bacteria. This inhaled SDT has great potential as a replacement for antibiotic treatment of drug-resistant bacterial pneumonia (Figure 14).

## 5. Conclusions and Perspectives for Future Work

Lung infections caused by bacteria continue to be a global problem of great concern. Since the discovery of penicillin in 1928, a series of antibiotics have been created that have saved the lives of countless patients. However, the extensive use of antibiotics has also caused the development of bacterial resistance, which is caused by multiple mechanisms such as the production of hydrolytic enzymes, alteration of targets of action, increased activity of efflux pumps, and biofilm formation. The search for antibiotic alternatives and the development of drug carriers lead to effective tools to address this issue.

Oral and intravenous antibiotic formulations have been used extensively in the clinic. Intravenous administration of drugs through systemic circulation avoids the first-pass effect and improves the bioavailability of the drug. However, intravenous formulations inevitably suffer from lack of pulmonary targeting and systemic toxicity in the treatment of pulmonary infections. Currently, bioactive nanoparticles prepared by nanotechnology to modify the size, surface properties, and hydrophobicity of drugs have improved the therapeutic effect. Multiple properties of nanoparticles can facilitate the delivery of antibiotics to the lungs. Common bioactive nanoparticles include liposomes, micelles, polymers, and inorganic nanoparticles. These nanoparticles have been extensively studied in order to effectively deliver antibiotics to infected cells or around bacteria, addressing the problem of bacterial resistance. Nanoparticles have been studied in the context of removal of bacteria that reside inside or outside of cells, and they have been used to deliver therapies to deal with the inflammation produced by bacterial infections. From a clinical perspective, much work remains to be completed before bioactive nanoparticles can transition from the research phase to clinical applications. Current challenges include the improvement of biocompatibility, biodegradability, and toxicity associated with nanoparticles.

Pulmonary inhalation drug delivery offers a new route of administration of therapies for pulmonary infectious diseases that directly addresses the issue of drug targeting. Inhalation administration reduces drug toxicity and leads to a more rapid onset of action compared to intravenous formulations. The process from drug inhalation to action is associated with a series of hurdles, and administration places difficult demands on the inhalation device. For instance, local administration of drugs that are toxic or readily cleared by the lungs may require a drug carrier. Many studies have also shown that the mucus barrier present in the lungs is a major challenge that may be overcome through the application of nanotechnology. In particular, in pneumonia, mucus secretion in the lungs increases, further preventing drugs from entering the cells of the lungs. Polymeric nanoparticles can penetrate this mucus, which makes them strong candidates for pulmonary drug delivery. Another key issue in this regard is the idea that hydrophilic drugs tend to be the best candidates for inhalation therapy; modification of hydrophobic drugs with nanoparticles can expand the scope of application.

In addition to drug delivery, nebulized inhalation combined with other means of treating pneumonia has shown good therapeutic results. With the help of inhaled acoustic sensitizers deep within in the lungs, SDT is able to remove recalcitrant bacteria, enabling highly effective non-invasive treatments. Recently, the use of phages for the delivery of pneumonia therapies has become an emerging modality that is related to nanotechnological solutions. Phage therapy has the advantages of high specificity, low drug resistance, and low incidence of side effects, driving the use of this tool in precision therapies. A number of antibiotic-alternative therapies have also received attention from the point of view of drug delivery modalities. The combination of multifunctional drug carriers as well as synergistic therapies is expected to provide new avenues for the treatment of bacterial pneumonia.
